# The influence of motor preparation on the processing of action-relevant visual features

**DOI:** 10.1038/s41598-019-47640-4

**Published:** 2019-07-31

**Authors:** Xavier Job, Mara Golemme, Joydeep Bhattacharya, Marinella Cappelletti, Jan de Fockert, Jose van Velzen

**Affiliations:** 1 Department of Psychology, Goldsmiths, University of London, London, UK; 2 Institut des Systèmes Intelligents et de Robotique (ISIR), Sorbonne Université, Paris, France; 3 Institute of Cognitive Neuroscience, UCL, London, UK

**Keywords:** Perception, Sensory processing, Human behaviour

## Abstract

Action preparation can facilitate performance in tasks of visual perception, for instance by speeding up responses to action-relevant stimulus features. However, it is unknown whether this facilitation reflects an influence on early perceptual processing, or instead post-perceptual processes. In three experiments, a combination of psychophysics and electroencephalography was used to investigate whether visual features are influenced by action preparation at the perceptual level. Participants were cued to prepare oriented reach-to-grasp actions before discriminating target stimuli oriented in the same direction as the prepared grasping action (congruent) or not (incongruent). As expected, stimuli were discriminated faster if their orientation was congruent, compared to incongruent, with the prepared action. However, action-congruency had no influence on perceptual sensitivity, regardless of cue-target interval and discrimination difficulty. The reaction time effect was not accompanied by modulations of early visual-evoked potentials. Instead, beta-band (13–30 Hz) synchronization over sensorimotor brain regions was influenced by action preparation, indicative of improved response preparation. Together, the results suggest that action preparation may not modulate early visual processing of orientation, but likely influences higher order response or decision related processing. While early effects of action on spatial perception are well documented, separate mechanisms appear to govern non-spatial feature selection.

## Introduction

Evidence for a tight coupling between action and perception has shown that perceptual processing can influence subsequent motor behaviour^1,2^. For instance, actions can be automatically primed by the visual properties of objects, such as their spatial location, orientation and size^3–6^. This coupling is also bidirectional, such that motor processing itself influences the processing of incoming perceptual information, i.e. *motor-visual priming*^7^. This is line with ideomotor theory, which broadly proposes that action selection involves perceptual representations of predicted action-effects^8–10^. Experimental findings have shown that preparing actions can affect stimulus processing, either facilitating or interfering with it (for reviews of facilitation versus interference effects see^10,11^). Stimuli are responded to faster if they are oriented in the same direction as a prepared grasping action^7^, or if the stimuli’s relative size is consistent with the prepared grasping action^12^. Similarly, stimuli are detected faster if they appear to rotate in the same direction as a planned manual object rotation^13^. These latter findings show that prepared actions can speed responses to action-relevant stimulus features, suggesting that perception of those features is facilitated.

However, speeded motor responses may not necessarily reflect changes to perception but may instead reflect changes to post-perceptual processes related to the decision/response. Unlike reaction times, signal detection measures of sensitivity (i.e. *d’*) reflect an observer’s ability to discern a sensory event from noise, thus providing a more unambiguous measure of perceptual processing^14^. Analysis of perceptual sensitivity, as well as accuracy in general is often precluded in motor-visual priming paradigms due to the use of relatively easy tasks of stimulus detection that garner error rates too low to be meaningfully analysed (although see Müsseler and colleagues^15^ who reported sensitivity measures for an interference effect). If effects of action on perception operate at the level of sensory perception, then preparing an action should influence an observer’s sensitivity to discriminate a perceptual feature that is action-relevant.

Here, we investigated whether action preparation influences perceptual sensitivity as well as response speed, when discriminating stimuli that share a feature with the prepared action. To investigate sensitivity to detect an action-relevant visual feature, participants were required to discriminate two briefly and simultaneously presented spatial frequency gratings as having the same or different orientations. Unlike previous studies^7,12,13^, to ensure sufficient error rates across participants, the difference between the two gratings was continually updated throughout the task depending on each participant’s performance. The key manipulation of interest was that, before the onset of the gratings, participants were cued to prepare one of two grasping actions, oriented either towards the right or the left. The orientation of the prepared grasp (leftward or rightward) and the orientation of the two gratings (both leftward or both rightward) could be either congruent or incongruent on any given trial. If action relevant features are prioritized at sensory processing stages, then reaction times and perceptual sensitivity to discriminate the stimuli should improve following congruent grasp cues, relative to incongruent grasp cues. If, however, action preparation mostly influences post-perceptual or response related processing stages, then only reaction times (and not perceptual sensitivity) should be affected by action-perception congruency.

While there is some evidence that preparation for different types of actions (e.g. grasping, reaching or pointing) can not only speed responses but also sharpen estimations of stimulus size^16^ and orientation^17^, these studies focused on priming feature perception at a more general dimensional level. These effects tap into how the planning and execution of the invariant characteristics of a movement (i.e. select *what* action should be executed such as a non-specific reach or grasp) affects the weighing of feature dimensions in visual search. In contrast, the current work focuses on how specific stimulus features can become flexibly tuned to the more fine-grained variant characteristics of a prepared action (i.e. prepare *how* an action should be accomplished such as a specific grasp orientation). In other words, perception may be biased not only in accordance with what overall action is prepared (e.g. grasping versus pointing), but perception may also be biased in accordance with how a specific action is accomplished (e.g. grasping at a specific location and degree of orientation).

Beyond behavioural measures of perceptual sensitivity, electroencephalography (EEG) can inform on the precise point in time of action-related modulations of visual processing in the brain. However, there are notably few EEG studies of motor-visual priming, with mixed patterns of results. One exception^18^ combined visual search tasks for size or luminance deviants with motor tasks of grasping and pointing while recording EEG. The typical behavioural pattern of motor-visual priming was found, such that pointing (compared to grasping) facilitated response times to detect luminance deviants and grasping (compared to pointing) facilitated response times to detect size deviants. While the early P1 component (70–130 ms) elicited by luminance deviants was enhanced during pointing compared to grasping, no such P1 modulation was observed for size deviants. In contrast, we recently showed an effect of grasp preparation on early visual ERPs elicited by task-irrelevant stimuli varying in their relative size^19^, such that N1 components (130–170 ms) elicited by larger stimuli were enhanced during power-grasp, compared to precision-grasp, preparation and N1 components elicited by smaller stimuli were further enhanced during precision-grasp, compared to power-grasp, preparation.

Here, we recorded EEG to investigate the time course with which action affects perceptual processing of stimulus orientation. If the effects of action planning on stimulus processing reflect biases in early visual processing, we expect that early ERP components elicited by the targets will be modulated by the congruency of the prepared action. However, action preparation may affect later processing stages such as motor preparation. Cued motor preparation is typically accompanied by prominent changes in the power of beta oscillations (13–30 Hz) over central electrode sites^20–22^, with neural sources in the contralateral pre-Rolandic ‘sensorimotor’ region^23^. While the exact functional role of beta band activity in cued movement tasks is not yet clear^22^, there is a general consensus that beta oscillations provide a reliable indicator of the onset of movement preparation, execution, as well as motor imagery^24–26^ and may reflect an active process promoting existing motor or cognitive states^27^. To date, no motor-visual priming studies have investigated beta oscillations. An exploratory approach was therefore taken to investigate whether action congruency influences sensorimotor beta oscillations.

## Experiment 1

### Methods

#### Participants

Forty-four adults (33 females, mean ± SD of age: 25 ± 4.96 years) participated in this experiment. This sample size was chosen to detect a medium effect size (e.g.${\eta }_{P}^{2}$ = 0.32) as observed in previous studies^17^ (for the main effect of congruency) with 0.85 statistical power to reject the null hypothesis given a critical threshold of 0.05 (two-tailed). All participants were right handed, with a mean laterality quotient^28^ of 90.62 (SD = 14.29), and reported normal or corrected to normal vision.

The Local Ethics Committee at Goldsmiths, University of London, approved all experimental protocols and all of the experiments adhered to the ethical guidelines presented in the 1964 declaration of Helsinki. All participants provided written informed consent before the beginning of each experiment and were debriefed at the end of each experiment as appropriate. Participants received either course credits or £10 for taking part in the study.

#### Stimuli and task

**Figure 1:**
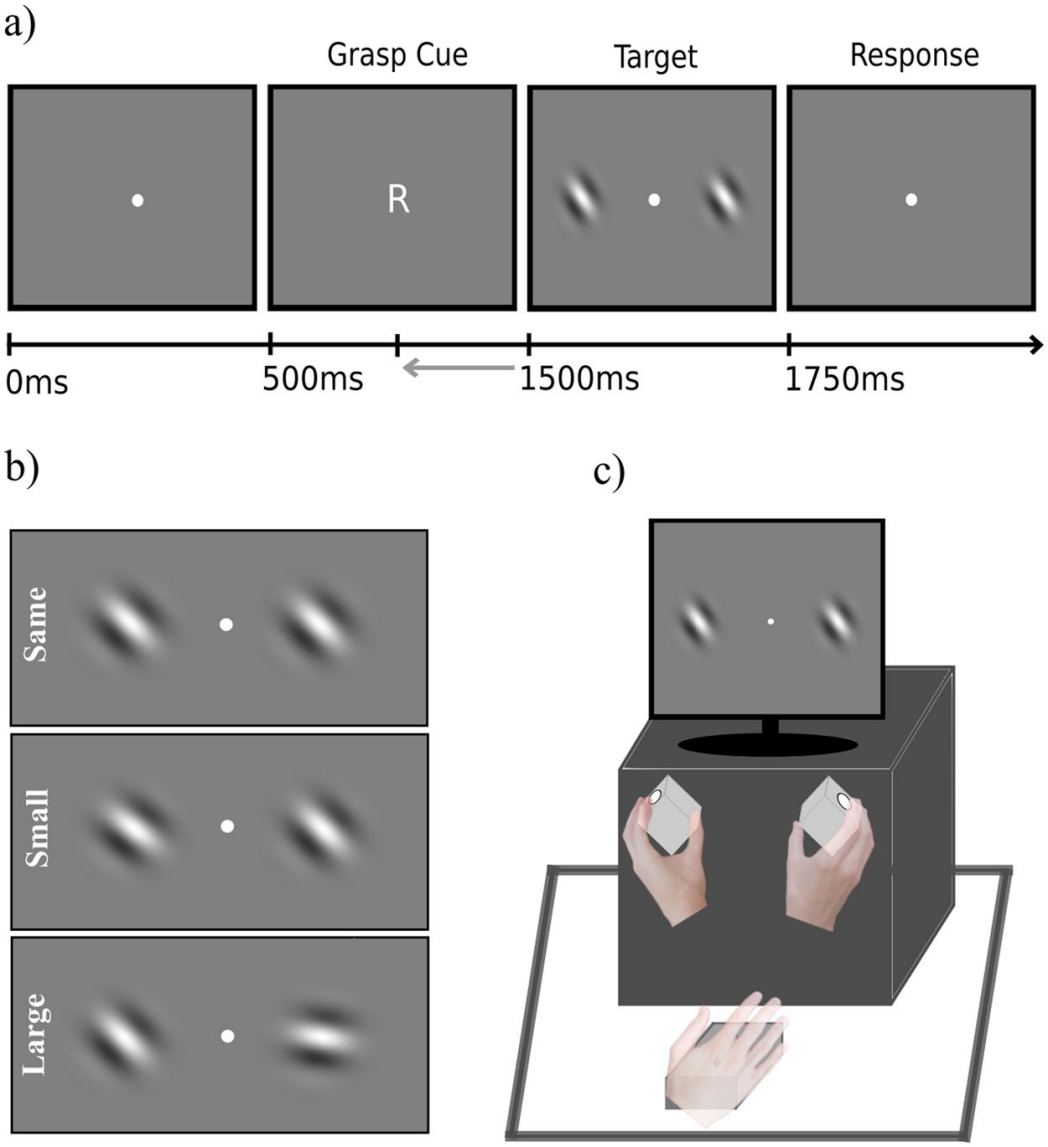
(**a**) Illustration of a typical trial. A rightward (‘R’) or leftward (‘L’) cue informs participants which grasp to prepare before spatial frequency gratings are presented. Participants execute the cued grasp if they perceive the gratings to be identical in orientation. (**b**) Examples of leftward oriented spatial frequency gratings either side of a fixation dot that are the same in orientation (upper panel), different by a small degree of orientation (middle panel) and different by a large degree of orientation (lower panel). (**c**) Illustration of the response device used. Two graspable cubes with response buttons mounted to the top left/right corners and angled at 45° and 315° to afford leftward/rightward oriented grasps. The lever device below initiated each trial when pressed and collected reaction times when released. Figure adapted from^29^.

Figure 1 (panel a) shows a schematic illustration of a trial (the figure has been adapted from the doctoral thesis of X.J.^29^). Spatial frequency gratings (4 cycles/degrees, contrast 100%, 4.5° eccentricity) were displayed on the horizontal meridian, on the left and right side (37% and 63% of the screen width, respectively) of a centred white fixation dot (visual angle 6°) on a grey background (see Fig. 1b). Stimuli were generated using MATLAB 2012a (64 bit) and presented using the Psychophysics Toolbox Version 3.0.8^30^.

Each trial was initiated by resting the right hand on a lever device. A movement cue (“R” or “L”) was then presented on the screen, indicating which grasp should be prepared with the right hand. Subsequently, two gratings were presented for 250 ms, which could be orientated either congruently or incongruently with the orientation of the prepared grasp, in equal amount. Both the orientation of the gratings (left/right) and the movement cue were varied randomly across trials. Participants were instructed to lift the hand from the lever device and execute the prepared leftward or rightward grasp only if the orientation of the two gratings was identical (i.e. 0° difference), otherwise participants continued to rest their hand on the lever device when the orientations of the gratings differed. Auditory feedback (200 Hz tone, duration = 100 ms) was presented following errors.

Responses were collected from a custom-built device (HxWxD: 30 cm × 30 cm × 32 cm) with two graspable cubes (5 cm × 5 cm × 5 cm) mounted on the upper left and right corners (see Fig. 1c). Depressible buttons were integrated into each cube. The cubes were tilted 45° and 315° such that in order to grasp them participants were required to orient their grasp either leftwards or rightwards to press the button with their index finger while the thumb made contact with underneath of the cube. The button recorded information about grasping responses. The lever device (4 cm × 9 cm × 11 cm) was placed in front of participants equidistant from the left and right grasping cubes. Resting the hand on the lever initiated each trial. The time elapsed between target onset and the time at which the lever was released (reaction time), and the time at which the grasp was made (movement time) were recorded.

Throughout the task, the orientation discrimination level was determined adaptively using Palamedes toolbox (http://www.palamedestoolbox.org) by a “four-correct-then-down/one-wrong-then-up” staircase procedure^31^. This ensured that perceptual discrimination was above chance, while allowing for adequate error rates for analysis. Furthermore, orientation discrimination was measured across two levels of perceptual difficulty by varying the degree of difference in orientation between the gratings to be relatively large (easy to discriminate) or relatively small (difficult to discriminate). In line with previous research^32^, six staircase steps were established with each step consisting of two orientation differences (‘small’ and ‘large’), that were always separated by 5°. Reference stimuli were either 5° or 60° from vertical 0° clockwise or counter clockwise. Therefore, the difference from the reference stimulus, either clockwise or counter clockwise, on any given trial could be: 22°/27° (staircase step 1), 19°/24° (step 2), 16°/21° (step 3), 13°/18° (step 4), 10°/15° (step 5), 7°/12° (step 6). Within each staircase step, one of two differences in degree could be presented (e.g. in staircase step 1, either 22° or 27° clockwise or counter clockwise) in order to manipulate the difficulty of the perceptual discrimination as either relatively large (e.g. 27° difference) or relatively small (e.g. 22° difference).

#### Procedure

Participants sat in a dimly lit room approximately 57 cm from a monitor, which was placed above the response device. Participants first completed a practice block of 20 trials, followed by six blocks of 86 trials with self-timed breaks between blocks. Trials were randomly selected from within the given staircase step. Trials could contain a rightward (50% of trials) or leftward grasp cue (50% of trials), followed by a congruently (50% of trials) or incongruently (50% of trials) oriented pair of gratings. Gratings were identical on 50% of trials and different by a small or large degree for 25% of trials each. Responses were therefore required on 50% of trials where the gratings were identical in their orientation. For half of the participants the cue-target interval was 1000 ms and for the other half the interval was 500 ms. The entire session lasted approximately 50 minutes.

#### Data analysis

For reaction time data, trials in which an incorrect grasping response was made were excluded as well as trials in which the reaction time fell outside ± 2.5 standard deviations of the mean (standard deviations were calculated for all trials, separately for each participant). This resulted in a mean of 2.15% (SD = 0.79) of trials rejected. A mixed Analysis of Variance (ANOVA) with a within-subjects factor of grasp congruency (congruent/incongruent) and a between-subjects factor of cue-target interval (1000 ms/500 ms) was used to analyse the reaction times.

For accuracy data, a mixed ANOVA with within-subjects factors of grasp congruency (congruent/incongruent) and orientation difference (small/large) and a between-subjects factor of cue-target interval (1000 ms/500 ms) was used to analyse the perceptual sensitivity values. Sensitivity (*d*’) was calculated by subtracting z-transformed false alarm rates (proportion of trials in which gratings that differ were responded to) from hit rates (proportion of trials in which gratings with 0° difference were responded to). Therefore d’ = Z(H) − Z(FA), where ‘H’ is the hit rate, ‘FA’ is the false alarm rate and ‘Z’ denotes the z-transformation. Z-transformed false alarm rates for small and large orientation differences were subtracted from the hit rates separately to calculate sensitivity values for these conditions.

A psychometric function was fitted to each participant’s data to obtain estimates of the underlying judgement noise during congruent and incongruent discriminations. The proportion of ‘same orientation’ responses was calculated for each degree of orientation difference. The data was then fitted with a model constructed from the differences of two cumulative Gaussian probability distributions^33,34^. This model was built for fitting data from simultaneity judgement tasks, in which participants judge whether two stimuli were simultaneously presented across a range of temporal delays. However, the model also applies to our task, in which participants judged whether two stimuli were tilted by the same angle across a range of orientation differences. Across all of the stimulus orientation differences the window of judged similarity is defined as the difference between a lower and upper decision boundary. The model parameter of interest was the standard deviation of the cumulative Gaussian for both boundaries (slope), reflecting the amount of sensory noise underlying participants judgements (akin to judgement uncertainty in the analysis of simultaneity judgement and temporal order judgement tasks^33^). These values were calculated for each participant and condition and entered into a mixed ANOVA with a within-subjects factor of grasp congruency (congruent/incongruent) and a between-subjects factor of cue-target interval (1000 ms/500 ms).

### Results

**Figure 2:**
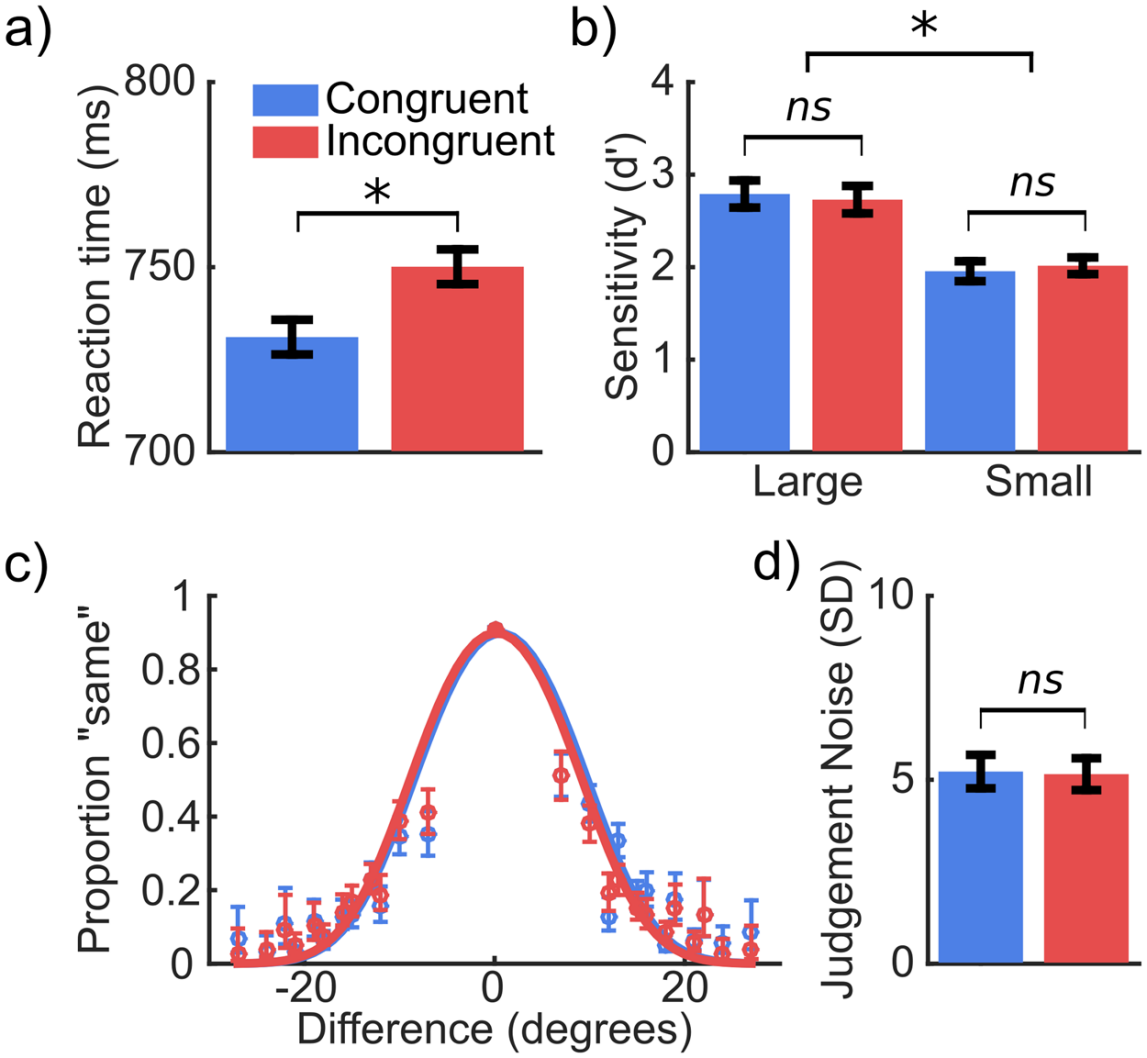
Behavioural results of Experiment 1. (**a**) Reaction times to discriminate gratings that had a congruent (blue) or incongruent (red) orientation with the prepared grasping action. Error bars show within-subjects 95% confidence intervals (CI)^63^. (**b**) Sensitivity to discriminate grasp congruent and incongruent gratings that had relatively large or small orientation differences. Error bars show within-subjects 95% CI. (**c**) Proportion of responses “same” for congruently (blue) and incongruently (red) oriented gratings across each level of orientation difference (left grating – right grating). The line shows the fit of a psychometric curve collapsed across participants, for illustrative purposes only - model parameters for individual participants were analysed. Error bars show 95% confidence intervals. (**d**) Estimation of the judgement noise (standard deviation of the cumulative gaussian for the lower and higher boundaries). Error bars show within-subjects 95% CI. *p < 0.01, *ns* p > 0.05. Figure adapted from^29^.

Grasp congruency significantly modulated reaction times (*F*(1, 42) = 16.38, *p* < 0.001, ${\eta }_{P}^{2}$ = 0.281), with faster responses for gratings with grasp congruent (*M* = 728 ms, *SE* = 16.14) compared to incongruent (*M* = 748 ms, *SE* = 16.47) orientations. Figure 2a shows the main effect of grasp congruency (the figure has been adapted from the doctoral thesis of X.J.^29^). No main effect of cue-target interval (*F*(1,42) = 0.03, *p* = 0.872) or interaction between grasp congruency and cue-target interval was observed (*F*(1,42) = 0.11 *p* = 0.738, ${\eta }_{P}^{2}$ = 0.003).

Orientation difference significantly modulated sensitivity (*F*(1,42) = 374.07, *p* < 0.001,${\eta }_{P}^{2}$ = 0.899) with lower sensitivity for stimuli with smaller differences in orientation (*M* = 1.99, *SE* = 0.04) compared to those with large differences (*M* = 2.76, *SE* = 0.06). Figure 2b shows the main effect of orientation difference. No main effects of grasp congruency (*F*(1,42) = 0.01, *p* = 0.991, ${\eta }_{P}^{2}$ = 0.001), or cue-target interval (*F*(1,42) = 1.06, *p* = 0.309, ${\eta }_{P}^{2}$ = 0.025) were observed for sensitivity. An interaction between orientation difference and cue-target interval was found (*F*(1,42) = 5.22, *p* = 0.027, ${\eta }_{P}^{2}$ = 0.111). The difference between small and large orientation differences was significant for cue-target intervals that were longer (*M* = 0.68, *SE* = 0.05) and shorter (*M* = 0.87, *SE* = 0.06), *t*(21) = 14.45, *p* < 0.001 and *t*(21) = 13.39, *p* < 0.001, respectively. The difference between long and short cue-target intervals was non-significant for orientation differences that were small (*M* = 0.001, *SE* = 0.07) and large (*M* = 0.18, *SE* = 0.11), *t*(42) = −1.59, *p* = 0.120 and *t*(42) = 0.01, p = 0.989, respectively. Grasp congruency did not interact with orientation difference (*F*(1,42) = 2.33, *p* = 0.135, ${\eta }_{P}^{2}$ = 0.053) or cue-target interval (*F*(1,42) = 0.03, *p* = 0.874, ${\eta }_{P}^{2}$ = 0.001). No significant three-way interaction was observed, *F*(1,42) = 0.18, *p* = 0.671, ${\eta }_{P}^{2}$ = 0.004. Means and standard deviations of the hit and false alarm rates across conditions can be found in the Supplementary Table 1.

For estimates of judgement noise derived from the fitting of the psychometric function, no main effect of grasp congruency (*F*(1,42) = 0.34, *p* = 0.559, ${\eta }_{P}^{2}$= 0.008) or cue-target interval (*F*(1,42) = 0.30, *p* = 0.587, ${\eta }_{P}^{2}$ = 0.007) was observed. No interaction between congruency and cue-target interval was observed either, *F*(1,42) = 0.02, *p* = 0.881, ${\eta }_{P}^{2}$ = 0.001. Figure 2c shows the proportion of “same” responses for each level of orientation difference presented. Estimates of judgement noise derived from fitting the psychometric function to the data are also shown in Fig. 2d.

### Discussion

In Experiment 1, participants responded when they perceived two spatial frequency gratings as identical in orientation (0° difference) with a pre-cued grasping action. Responses were faster when the gratings were oriented in the same direction as a prepared grasping action (congruent), compared to the opposite direction (incongruent). This is in line with previous findings that grasping actions are initiated faster when signalled by a congruently oriented stimulus^7,13^. However, no differences were observed between grasp congruent and incongruent discriminations in perceptual sensitivity (*d’*) or estimates of judgement noise for both cue-target intervals of 1000 ms and 500 ms. Without an effect of motor preparation on perceptual sensitivity, it is difficult to determine whether the congruency effect in the response times was due to motor-visual priming, visuo-motor priming, or a combination of the two. This was directly addressed in a dual-task paradigm in Experiment 3.

The difficulty of the perceptual discrimination was also manipulated to be relatively easy (large orientation differences) or relatively difficult (small orientation differences). If effects of action on perception depend on the perceptual resources currently available, then motor-visual priming effects may vary as a function of difficulty. Previous studies are inconsistent regarding perceptual difficulty in motor-visual priming. For example, some have shown that effects of action on perception in a task of visual search can vanish at larger set sizes^35,36^, suggesting that action is only capable of influencing perception when sufficient perceptual resources are available. However, more recently the same effects of action preparation on orientation change detection were observed across three levels of difficulty^17^, suggesting no influence of perceptual difficulty. Here the congruency of the prepared action did not interact with the discrimination difficulty, even though the difficulty of the perceptual discrimination had a strong effect on performance, with higher sensitivity for larger, compared to smaller orientation differences. However, given that participants were instructed to respond only when the gratings were the same in orientation, any effect of difficulty on reaction times (where we did find evidence for motor-visual priming) was precluded. To address this, in Experiment 2 participants were instructed to respond only when the stimuli were *different* in orientation. If the difficulty of the perceptual discrimination influences motor-visual priming, then the reaction time advantage for congruent (compared to incongruent) stimuli may be affected by discrimination difficulty.

## Experiment 2

### Methods

#### Participants

Twenty-two adults (16 females, mean ± SD of age: 24 years ± 3.97 years) took part in the experiment. All participants were right handed, with a mean laterality quotient^28^ of 92.02 (SD = 14.24), and reported normal or corrected to normal vision. Ethical approval, informed consent, debriefing, and compensation were the same as in Experiment 1.

#### Stimuli and task

The stimuli and task were identical to those of Experiment 1, except that participants were instructed to respond only when the grating stimuli were different in orientation, and to make no response when they were identical. The cue-target interval was fixed at 1000 ms as the factor of cue-target interval in Experiment 1 did not interact with the factor of congruency.

#### Data analysis

Reaction time data and sensitivity values (*d’*) were analysed in separate repeated-measures ANOVAs with factors of grasp congruency (congruent/incongruent) and orientation difference (large/small). A mean of 3.6% (SD = 0.90) of trials were rejected for either having reaction times that fell outside ± 2.5 SD of the mean (calculated separately for each participant pooled across conditions) or where a grasping error was made.

### Results

In the reaction times, a main effect of grasp congruency was present (*F*(1,21) = 7.14, *p* = 0.014, ${\eta }_{P}^{2}$ = 0.254), with faster reaction times for grating stimuli that were congruent with the cued grasp (*M* = 660 ms, *SE* = 16.98), compared to incongruent 673 ms, SE = 17.86). A main effect of orientation difference was also observed (*F*(1,21) = 52.85, *p* < 0.001, ${\eta }_{P}^{2}$ = 0.715), with faster reaction times to gratings with large differences (*M* = 652 ms, *SE* = 17.11) compared to small (*M* = 682 ms, *SE* = 17.68). No interaction between grasp congruency and orientation difference was found (*F*(1,21) = 0.04, *p* = 0.839, ${\eta }_{P}^{2}$ = 0.002).

Orientation difference modulated perceptual sensitivity (*F*(1,21) = 221.06, *p* < 0.001, ηp2 = 0.913), with greater sensitivity for large differences (*M* = 2.58, *SE* = 0.07) compared to small (*M* = 1.85, *SE* = 0.04). Grasp congruency did not modulate sensitivity (*F*(1,21) = 0.001, *p* = 0.982, ${\eta }_{P}^{2}$ = 0.001) and did not interact with orientation difference (*F*(1,21) = 0.99, *p* = 0.331, ${\eta }_{P}^{2}$ = 0.045). Means and standard deviations of the hit rates, false alarm rates, sensitivity and reaction times across experimental conditions can be found in the Supplementary Table 2.

### Discussion

Experiment 2 required participants to discriminate two spatial frequency gratings as the same or different in orientation, following a cue to prepare a grasping action, just as in Experiment 1. However, a pre-cued grasping action was executed only when participants perceived the stimuli to be different from each other in orientation, rather than the same. This simple change to the task instructions allowed for the measurement of the reaction time congruency effect as a function of difficulty in the task. While reaction times were affected by grasp congruency and the difficulty of the discrimination, there was no interaction between these factors.

The finding that sensitivity to discriminate the target stimuli was unaffected by the congruency of the prepared grasp in Experiments 1 and 2 suggests that the reaction time advantage for grasp-congruent stimuli originates in processes other than perceptual sensitivity. To further investigate the time course of the action-congruency effect, in Experiment 3 we used electrophysiology to identify the stage at which action may affect processing. We predicted that if the reaction time effect observed in Experiments 1 and 2 indeed reflects a modulation of post-perceptual processing, then later rather than earlier EEG effects related to the targets should be associated with congruency of the prepared action.

The congruency effect observed for reaction times in Experiments 1 and 2 (i.e. faster responses to gratings oriented in the same direction as a pre-cued grasping action) could conceivably result from stimulus-response priming, rather than an effect of action planning on stimulus processing: although participants prepared an action that could either be congruent or incongruent with the upcoming visual stimulus, the required action following target presentation was also either congruent or incongruent with the target, leaving open the possibility that the congruency effect was partly driven by stimulus-response priming. To rule out this possibility, Experiment 3 was adapted to be dual-task, following previous motor-visual priming studies^7,13,37^. Participants discriminated the grating stimuli with a key press response, then subsequently executed the prepared grasping action following a ‘GO’ stimulus. If the previous advantage for congruently oriented grating reflects motor-visual effects rather than the opposite, then the effect should also be present for key presses. As no interactions between discrimination difficulty and grasp congruency were observed for Experiments 1 and 2, difficulty was no longer a factor in the design of Experiment 3.

## Experiment 3

### Methods

#### Participants

Twenty-four adults (18 females, mean ± SD of age: 27 years ± 4.32 years) participated in the experiment. All participants were right handed, mean laterality quotient^28^ = 92.18, *SD* = 14.18, and reported normal or corrected to normal vision. Ethical approval, informed consent, and debriefing were the same as in Experiment 1. Each participant received £20 for taking part.

#### *Stimuli* & task

**Figure 3:**
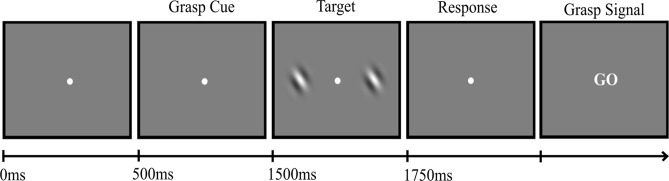
Experiment 3 trial procedure. Participants fixated on the central dot throughout the task. Grasp cues (1000 Hz/400 Hz tones) informed participants which grasp to prepare before spatial frequency gratings were presented. Participants then discriminated the gratings as the same or different in orientation from each other by pressing the ‘S’ or ‘D’ keys, respectively. A grasp signal (“GO”) was presented 200 ms after the key press, which signalled the execution of the cued grasp. Figure taken from^29^.

The stimuli and task were identical to Experiment 1, except for the following. The movement cues consisted of high (1000 Hz) and low (400 Hz) frequency tones, and the cue-tone mapping was counterbalanced across participants. Participants were instructed to respond to the grating stimuli by pressing the ‘S’ or ‘D’ keys on a keyboard with the middle and the index finger of their left hand if they perceived the stimuli to be the same or different in orientation, respectively (see Fig. 3, which has been adapted from the doctoral thesis of X.J.^29^). Following this, a signal to execute the grasping movement was presented as the word ‘GO’ in the centre of the screen. The manipulation of difficulty (relatively small or large orientation differences) was removed from the design for Experiment 3 as this factor did not significantly interact with grasp congruency in Experiments 1 and 2. Therefore, 50% of trials consisted of relatively small orientation differences (either 22°, 19°, 16°, 13°, 10° or 7° depending on the level of the adaptive staircase) and 50% consisted of 0° orientation differences. The cue-target interval was 1000 ms.

#### Procedure

In addition to the procedure outlined for Experiments 1 and 2, the length of the entire session was extended to approximately two hours to include preparation of the EEG recording.

#### Behavioural data analysis

Separate ANOVAs with grasp congruency (congruent/incongruent) as a within-subject factor were used to analyse sensitivity values (*d*’), estimated of judgement noise and reaction times of the grating discrimination. For reaction time data, 3.65% (*SD* = 0.03) of trials were rejected for either having reaction times that fell outside ± 2.5 *SD* of the mean or because they corresponded to incorrect grasping.

#### *EEG recording*, *pre-processing and analysis*

EEG was recorded using a BioSemi ActiveTwo amplifier from 64 Ag–AgCl electrodes placed according to the extended 10–20 system and sampled at 1024 Hz; EEG signals were later down-sampled offline to 512 Hz. The electrodes were referenced to the average of electrodes placed on the left and right earlobes. The vertical and horizontal EOGs were recorded in bipolar fashion in order to monitor vertical (i.e. blinks) and horizontal (i.e. saccades) eye movements, respectively. Offline pre-processing and analysis of the EEG data was conducted using a combination of EEGLAB version 13.4.4b^38^ and FieldTrip toolboxes^39^.

For the analysis of the grating stimulus evoked potentials, continuous EEG data were divided into 700 ms epochs locked to the onset of the stimulus including a 100 ms pre-stimulus baseline. Epochs including voltages exceeding + and/or − 100μV, well as epochs with discrimination or grasping errors, were automatically rejected prior to analysis. Eye-blink artefacts were corrected for using Independent Component Analysis. The mean amplitudes of ERP components within pre-defined time windows were extracted for analysis. The mean amplitude between 70 and 110 ms post probe onset was extracted as the P1 mean amplitude^40^. The mean of amplitudes between 130–170 ms post probe onset was extracted as the N1 mean amplitude^40^. Mean amplitudes were extracted from electrode sites PO7 and PO8, which elicited the largest ERPs as observed in scalp maps of averages over all conditions. The difference between the mean P1 and N1 values was computed to obtain a mean peak-to-peak amplitude measure of the N1 component.

For the ERP analysis, the mean peak-to-peak amplitudes of the N1 component were analysed in a 2 × 2 repeated measures ANOVA with factors of grasp congruency (congruent/incongruent) and electrode hemisphere (PO7/PO8).

For the time-frequency analysis of beta power (13–30 Hz) following the grating stimuli, continuous EEG data were divided into 1000 ms epochs including a 300 ms pre-stimulus baseline. Time frequency representations of individual trials were then calculated using Morlet wavelet analysis with a wavelet width that linearly increased from 3 to 8 with the frequency range. Trials were then averaged for each condition and normalised to the pre-stimulus baseline period (−300ms to 0 ms).

For the statistical analysis of grating stimulus-locked beta power, non-parametric cluster permutation was used^41^. This approach to the analysis of multidimensional neuroimaging data extracts spatiotemporal regions showing significant differences between conditions or groups without any a priori assumptions of spatial regions or time windows. It therefore identifies effects that are robust within a cluster of electrodes/time points, rather than highly significant on one dimension (i.e. a single electrode and/or time point). The method is robust against Type I error as this is intrinsically controlled for by evaluating only the maximum cluster-level statistics under the null hypothesis.

The following steps were taken to identify significant clusters: 1) dependent samples t-statistics comparing grasp congruent and incongruent data were gathered for each of the samples in the multidimensional data structure; 2) t-statistics above a p-value threshold (*p* < 0.05) were then gathered; 3) Neighbouring data points exceeding the threshold were found; 4) The t-statistics were summed to calculate the cluster level statistic; 5) The maximum cluster statistic under its permutation distribution (shuffled data), derived from the test statistics obtained from the dependent samples t-tests based on 1000 random permutations, was evaluated. The cluster-level significance threshold was set at the two-tailed level of 0.025. Electrodes had an average of 6.6 neighbouring electrodes. Finally, dependent-samples t-tests were run on beta power values at cluster electrodes/time points comparing congruent and incongruent conditions.

### Results

#### Behavioural data

For reaction times a main effect of grasp congruency was present (*F*(1,23) = 8.16, *p* = 0.009, ${\eta }_{P}^{2}$ = 0.262), with faster reaction times for grating stimuli that were congruent with the cued grasp (*M* = 822 ms, *SE* = 21.31), compared to incongruent (*M* = 842 ms, *SE* = 20.85).

Grasp congruency did not modulate sensitivity (*d’)* to discriminate the stimuli (*F*(1,23) = 0.15, *p* = 0.699,${\eta }_{P}^{2}$ = 0.007). The proportion of hits and false alarms for each condition can be found in the Supplementary Table 3. Estimates of judgement noise did not significantly differ between congruent and incongruent conditions (*F*(1,23) = 1.13, *p* = 0.300, ${\eta }_{P}^{2}$ = 0.047).

#### Visual evoked potentials

**Figure 4:**
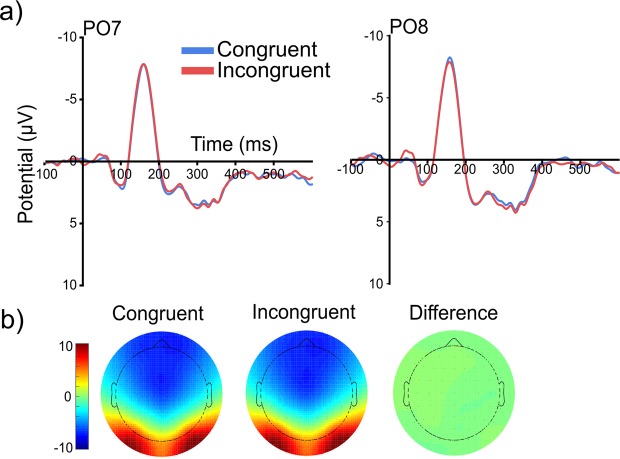
(**a**) Grand average event-related potentials (ERPs) elicited by the grating stimuli (onset = 0 ms) presented following the cues instructing the preparation of congruently oriented (blue) or incongruently oriented (red) reach-to-grasp actions. (**b**) The scalp maps show the distribution of the N1 component peak-to-peak amplitude (μV) elicited by gratings presented following congruent grasp cues (left scalp map) and incongruent grasp cues (centre scalp map) as well as the difference (right scalp map). Figure adapted from^29^.

Figure 4 shows the grand average event-related potentials (ERPs) elicited by the grating stimuli (the figure has been adapted from the doctoral thesis of X.J.^29^). For the N1 mean peak-to-peak amplitude, no main effect of grasp congruency (*F*(1,23) = 0.05, *p* = 0.820, ${\eta }_{P}^{2}$ = 0.002), or electrode hemisphere (*F*(1,23) = 0.13, *p* = 0.717, ${\eta }_{P}^{2}$ = 0.006) was found, nor an interaction between grasp congruency and electrode hemisphere (*F*(1,23) = 0.277, *p* = 0.604, ${\eta }_{P}^{2}$ = 0.012).

#### *Stimulus-locked beta power (13–30* *Hz)*

**Figure 5:**
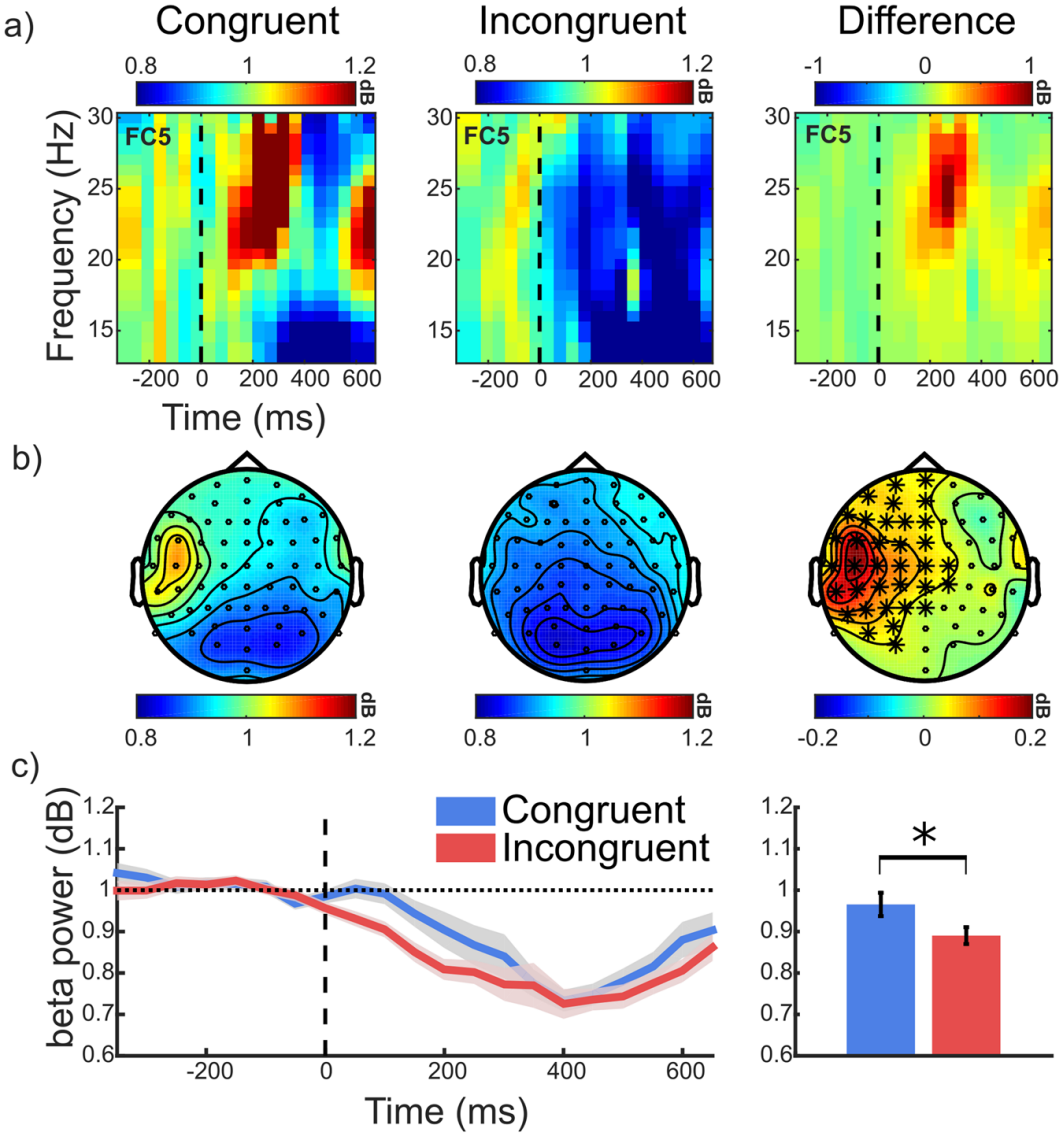
Grand averaged stimulus-locked power in the beta band (13–30 Hz). (**a**) Time-frequency plots at a representative electrode (FC5) locked to stimulus onset (dashed line). (**b**) Scalp maps of beta power at significant time points (0–200 ms). Significant cluster electrodes are highlighted. (**c**) Beta power across time (left panel) averaged over significant cluster electrodes. Shaded areas show +/− SEM. The bar graph shows the averaged power at significant cluster electrode sites and time points for congruent (blue) and incongruent (red) conditions. Error bars show +/− SEM. Figure adapted from^29^.

Figure 5 shows the power in the beta band (13–30 Hz) following the grating stimuli that were oriented congruently or incongruently with the prepared grasping action (the figure has been adapted from the doctoral thesis of X.J.^29^). Higher beta power was observed in the congruent condition at left sensorimotor electrode sites (ipsilateral to the hand used to make the orientation discrimination key press). A significant positive cluster reflected this difference over sensorimotor electrode sites (significant cluster electrodes are highlighted with asterisks, *p* < 0.025) from stimulus onset until approximately 200 ms post-stimulus onset. A dependent-samples t-test comparing mean beta power across cluster electrodes/time points for congruent and incongruent conditions showed that higher power (relative to baseline) was observed for the congruent (*M* = 0.97 *SE* = 0.03) compared to incongruent (*M* = 0.89, *SE* = 0.03) condition (*t*(23) = 3.12, *p* = 0.005).

### Discussion

Experiment 3 aimed to further investigate whether or not the reaction time advantage for congruently oriented stimuli is associated with changes in early processing in the visual cortex. To do this, early event-related potentials elicited by the target stimuli during congruently or incongruently oriented grasping actions were compared. No differences in the amplitude of early visual ERPs were observed. We therefore do not find evidence that the reaction time advantage observed for the action-congruent stimulus feature is accompanied by modulations in early visual processing.

Instead, greater power of beta band (13–30 Hz) oscillations was observed over sensorimotor electrode sites in the hemisphere ipsilateral to the hand used to make the orientation discrimination key press response. Modulations of power in the beta band over sensorimotor sites are commonly observed during preparation of motor responses^24,25^. Studies typically report event-related *desynchrony* (ERD) in the hemisphere contralateral to the hand used to execute the movement and event-related *synchrony* (ERS) in the hemisphere ipsilateral to the hand used^42^. ERS is observed when the behaviour of large numbers of neurons synchronises^43^ and most likely requires coherent activity of cell assembles over at least several square centimetres^44^. Initially, beta ERS was thought to reflect the activation state of the sensorimotor system with ERS reflecting an ‘idle’ state and ERD an activation state^20^. This idea was supported by combined EEG/fMRI studies linking ERD to cerebral activation^45–47^. More recent theories suggest that ERS may reflect a maintenance of the current sensorimotor or cognitive state^27,48^, or possibly the dynamics of decision making processes^49,50^.

Our finding of increased beta power in the action-perception congruent condition may suggest that the left sensorimotor cortex underwent greater suppression following a congruent compared to incongruent imperative stimulus. This is consistent with the imperative stimulus requiring a left-hand key press, hence right hemisphere dominance. It is therefore possible that following congruently oriented stimuli, the left sensorimotor cortex was more effectively suppressed compared to incongruently oriented stimuli. Greater suppression of the left sensorimotor cortex (i.e. right hand) may result in speeded responses in the congruent condition, compared to the incongruent condition. Alternatively, the ERS of beta could reflect processing related to stimulus expectancy, as some reports show ERS in the anticipation of stimuli requiring a motor response at and even before cue onset^51–53^. This ‘anticipatory’ ERS is also absent in studies where the cue-target interval was variable, thus discouraging temporal anticipation of the stimuli^54,55^. The beta ERS we observed with a fixed cue-target interval occurred around stimulus onset until 200 ms post onset, consistent with previous findings of ‘anticipatory’ modulation of beta synchrony.

Experiment 3 also aimed to rule out an alternative explanation of the findings of Experiments 1 and 2, that the reaction time advantage reflected visual-motor rather than motor-visual priming. The paradigm was adapted into a dual-task such that the orientation discrimination was made using a response mode that did not contain a congruency with pre-cued action (key press), and the pre-cued action was instead signalled by a subsequent ‘GO’ stimulus. The reaction time advantage of congruently oriented targets was again observed in Experiment 3. Therefore, the effects observed in Experiments 1 and 2 indeed reflected motor-visual priming rather than the reverse. This is in line with previous motor-visual priming studies whereby reaction time effects were maintained even when the effector used to signal the perceptual decision exhibits no congruency with the prepared action^7,12,13^.

## General Discussion

Recent theories of action and perception suggest bidirectional links between these two domains. While there is a wealth of evidence that the visual properties of objects can bias actions (visual-motor priming), much less is known about how actions can influence visual processing (motor-visual priming). Some reports have demonstrated that cueing a simple manual action can speed responses to stimuli that share a perceptual feature with the action^7,13^, suggesting visual processing is biased by prepared actions. However, speeded responses may reflect changes in early perceptual processing, and/or decision and response related processing. In three experiments, we investigated if planned actions influence reaction times as well as perceptual sensitivity (*d’*) to discriminate visual stimuli that share a feature with a prepared action. Participants discriminated the orientation of two spatial frequency gratings as the same or different in orientation. The difference between the gratings was continuously adjusted according to participants’ performance throughout the task, in order to ensure the task produced adequate error rates. The effect of cueing an oriented grasping action prior to stimulus onset was investigated across two levels of perceptual difficulty as well as two cue-target intervals. Finally, EEG was recorded in order to investigate whether early event-related potentials elicited by the target stimuli were modulated by action intentions.

In line with previous findings, we observed faster responses to stimuli that were oriented in the same direction as an oriented grasping action. However, perceptual sensitivity (*d’*) to discriminate the stimuli, as well as estimates of sensory noise, were unaffected by the prepared action. This was the case across two levels of discrimination difficulty that were continuously adapted to participants’ performance across the task as well as across varying cue-target intervals. The reaction time advantage observed for congruently oriented gratings was also unaffected by the difficulty of the discrimination and was not accompanied by modulations of early event-related potentials elicited by the stimuli. Instead the reaction time advantage for congruent stimuli was associated with a modulation of beta band (13–30 Hz) synchrony over sensorimotor electrode sites, indicative of response related processing.

It is interesting to note that many previous studies have shown that preparing a variety of spatially guided movements (eye-movements, reaching and pointing) results in modulations of early ERP components (P1/N1), known to index sensory processing in extrastriate visual cortex. For example, P1 and N1 components elicited by task-irrelevant visual stimuli are enhanced if they appear at the goal location of planned eye-movements^56,57^, as well as at effector and goal locations of reaching movements^58–60^. These studies have typically measured neural responses to completely task-irrelevant stimuli in order to index sensory processing across space. It is conceivable that the requirement for a response in our task could discourage early sensory selection until a later stage after sensory perception, resulting in no differences in the early ERP components elicited by the stimuli. The question of how the wider context of the task influences modulations of early sensory ERP components is an intriguing one. Indeed some findings have shown that simply changing the task instructions to emphasise either the goal location of a movement or the effector to be used can profoundly alter the pattern of early ERP component effects^58^. Such findings suggest that effects of action on sensory processing may be dependent on a range of contextual factors. Actions rarely occur in isolation, and the ways in which aspects of the surrounding context, including task-demands, behavioural goals as well as practice and familiarity, affect action-perception coupling remain key areas of investigation.

The experiments reported here are not the first to report null effects of action preparation on behavioural tasks of perceptual accuracy. Another previous study reported three experiments in which left/right pointing movements were cued and accuracy to discriminate a visual target that appeared at the goal of the movement or at a different location was measured^61^. They found that the perceptual report was unaffected by the direction of the prepared action, across four different stimulus-onset-asynchronies. However, studies investigating the priming of more general feature dimensions (e.g. size vs. location or orientation) by cueing qualitatively different actions (e.g. grasping vs. pointing) have shown effects on accuracy and perceptual sensitivity. For example cueing a grasping action (compared to pointing) improved sensitivity to discriminate stimulus orientation^17^ as well as size^16,37^. Those studies focused on priming perceptual features at a more general *dimensional* level during the preparation of qualitatively different types of actions (e.g. grasping or pointing). In contrast we focused on the priming of *specific* features consistent with the characteristics of a prepared action (e.g. leftward or rightward oriented grasping action). Furthermore, previous studies have typically cued the same action within experimental blocks, so participants have prior knowledge of the action before trial onset. Improved sensitivity may therefore reflect a more general configuring of attentional sets for a feature dimension across a block, rather than a more flexible reallocation of perceptual resources to meet the requirements of current action planning. In our design, both the cued action and the stimulus congruency were varied randomly across trials, precluding any prior knowledge of action/stimulus congruency. Further research should elucidate whether cueing qualitatively different actions (e.g. grasping vs. pointing) influences visual sensitivity to discriminate congruent feature dimensions (e.g. size vs. location targets) without participants’ prior knowledge of the action and/or target stimulus feature before trial onset.

As expected, the difficulty of the perceptual discrimination had a large effect on reaction times, accuracy, and sensitivity in Experiments 1 and 2, such that responses were faster, and discrimination was more sensitive for larger orientation differences (easy to discriminate) versus smaller orientation differences (difficult to discriminate). However, no interactions were observed between the difficulty of the perceptual discrimination and the congruency with the prepared grasp. This suggests that any coupling between perception and action observed here is not affected by the difficulty of the perceptual discrimination. This is in contrast to findings that increasing the number of items in visual search tasks influences motor-visual priming effects with effects vanishing at larger set sizes^36,62^. This initially suggested that the processing resources shared by action and perception are to some extent capacity limited, as at larger set sizes there are insufficient resources for actions to further enhance stimulus processing. However, in one previous study^36^, motor-visual priming was also absent at very low set sizes, suggesting that behaviourally relevant stimuli are facilitated only when the task is appropriately challenging. Furthermore, in line with our findings, previous work^17^ has also found the same effects of preparing a grasping (versus pointing) action on orientation change detection across three levels of difficulty.

Congruency across the experiments was defined as the overlap between the orientation of the stimuli (leftward/rightward) and the prepared action (leftward/rightward). However, the action involved not only a grasp orientation (leftward/rightward oriented) but also a grasp location (left/right response device). The orientation and location of the action was always consistent, such that leftward and rightward oriented grasps were performed on the response devices positioned on the left and right, respectively. It is therefore not possible to distinguish whether the congruency effect is driven by an overlap with grasp orientation or grasp location. The relative contribution of an action’s spatial and featural components represents key area of investigation for future studies. For example, an additional congruency between the spatial (i.e. grasp location) and featural (grasp orientation) components of the action may reveal their relative contributions to motor-visual priming.

Finally, given that the reaction times to the grating stimuli in Experiments 1 and 2 were gathered from the execution of the cued movement itself, it is possible that perception of the leftward/rightward-oriented gratings facilitated the action, rather than the reverse. However, the results of Experiment 3 rule out this alternative explanation, as the same effect was observed when the orientation discrimination was made using a key press rather than the cued action itself. This is in line with many previous motor-visual priming studies that have repeatedly shown reaction time effects very similar to ours which are maintained even when the effector used to signal the perceptual decision exhibits no congruency with the prepared action^7,12,13^.

## Conclusions

Overall, we present a typical motor-visual priming effect across three experiments whereby visual stimuli are detected faster if they are oriented in the same direction as a prepared grasping action, compared to stimuli oriented in an opposite direction. Such effects are often interpreted as reflecting a selection mechanism working to prioritise the processing of action-relevant information. Previous research has shown that preparing spatially directed movements (e.g. eye-movements, reaching or pointing) influences very early visual processing, as indexed by early ERP components (P1/N1), thus demonstrating the tight coupling between perceptual and action systems. However, much less is known about how actions influence the processing of non-spatial visual features, beyond the reaction time effects typically observed. We investigated this further and found that in our experiments, perceptual sensitivity to discriminate the visual stimuli was unaffected by action preparation. Furthermore, the reaction time advantage was not reflected by a modulation of early ERP components (P1/N1) elicited by the visual stimuli. Instead the reaction time effect was reflected by synchronization of beta band (13–30 Hz) oscillations over sensorimotor areas shortly after stimulus onset. Together, these finding suggest that non-spatial motor-visual priming effects observed may not reflect modulations of early stages of visual processing, but the effects may in fact operate at either later stages of visual processing, or at decision and/or response related stages. The early effects of action on perception, that are well documented for spatial selection, appear to not generalise to non-spatial feature selection.

## Supplementary information

Supplementary Information

## Data Availability

The data generated and analysed are available from the corresponding author on reasonable request.
