# Supplementary material for: The influence of motor preparation on the processing of action-relevant visual features
Source: Sci Rep. 2019 Jul 31;9:11084. doi: 10.1038/s41598-019-47640-4 (PMC6668476; doi:10.1038/s41598-019-47640-4)
Supplement: Supplementary file 1 — Supplementary Information [file 41598_2019_47640_MOESM1_ESM.docx]

**The influence of motor preparation on the processing of action-relevant visual features.**

Xavier Job[[1]](#footnote-1) a,b, Mara Golemme^a^, Joydeep Bhattacharya ^a^, Marinella Cappelletti ^a,c^, Jan de Fockert ^a^, Jose van Velzen ^a^

^a^ Department of Psychology, Goldsmiths, University of London, UK

^b^ Institut des Systémes Intelligents et du Robotique (ISIR), Sorbonne Université, Paris, France

^c^ Institute of Cognitive Neuroscience, UCL, London, UK

**Supplementary Information 1.**

For Exp. 1 the percentage of trials rejected did not significantly differ between congruent and incongruent trials (*F*(1, 42)=1.78, *p*=.189, =.041), or the two Cue-target intervals (*F*(1, 42)=.843, *p*=.364, =.020) and no significant interaction was found either (*F*(1, 42)=.003, *p*=.958, =.000). In Exp. 1, there was no factor of Orientation Difference (large/small) for the reaction time analysis, as participants were instructed to respond only when the stimuli were the same. For Exp. 2 the percentage of trials rejected was not significantly different between congruent and incongruent trials (*F*(1, 21)=1.80, *p*=.194, =.079), or between small and large differences (*F*(1, 21)=.496, *p*=.489, =.023) and no significant interaction was found either (*F*(1, 21)=.196, *p*=.662, =.009). Finally, for Exp. 3 no significant difference in the number of rejected trials was found between congruent and incongruent stimuli (*t*(23)=.901, *p*=.376).

**Supplementary Table 1.**

| **Table 1. Mean reaction times (ms), proportion of Hits and False Alarms, Sensitivity (*d’*) and judgement noise (SD) values for Experiment 1. Standard deviations in parentheses** | | | | | |
| --- | --- | --- | --- | --- | --- |
|  | | **Cue-target interval 1000ms** | | **Cue-target interval 500ms** | |
|  | **Difference** | **Congruent** | **Incongruent** | **Congruent** | **Incongruent** |
| **Reaction Times** | **0°** | 730 (91.05) | 750 (95.56) | 727 (121.00) | 744 (121.39) |
| **Hits** | **0°** | .90 (.06) | .91 (.05) | .91 (.05) | .90 (.05) |
| **False Alarms** | **Small** | .29 (.06) | .27 (.07) | .29 (.08) | .27 (.06) |
|  | **Large** | .10 (.04) | .11 (.05) | .08 (.05) | .09 (.06) |
| **Sensitivity (d’)** | **Small** | 1.94 (.35) | 2.03 (.30) | 1.97 (.37) | 2.00 (.30) |
|  | **Large** | 2.70 (.45) | 2.64 (.36) | 2.88 (.51) | 2.82 (.58) |
| **Judgement Noise (SD)** | **> 0°** | 5.35 (1.43) | 5.26 (1.11) | 5.10 (1.58) | 5.15 (1.42) |

**Supplementary Table 2.**

| **Table 2. Mean reaction times (ms), proportion of Hits and False Alarms (FA) and Sensitivity (*d’*) values for Experiment 2. Standard deviations in parentheses** | | | |  |
| --- | --- | --- | --- | --- |
|  | | **Difference** | **Congruent** | **Incongruent** |
| **Reaction Times** | | **Small** | 675 (80.13) | 689 (87.21) |
|  |  | **Large** | 645 (81.43) | 658 (81.37) |
| **Hits** | | **Small** | .93 (.05) | .92 (.05) |
|  |  | **Large** | .95 (.03) | .93 (.05) |
| **False Alarms** | | **0°** | .19 (.09) | .19 (.07) |
| **Sensitivity (d’)** | | **Small** | 1.83 (.34) | 1.87 (.28) |
|  |  | **Large** | 2.60 (.44) | 2.56 (.38) |

**Supplementary Table 3.**

| **Table 3. Behavioural data for Experiment 3. Means and standard deviations (St. Dev).** | | | | |
| --- | --- | --- | --- | --- |
|  | **Congruent** | | **Incongruent** | |
| **Reaction Times** | 822 | (104) | 842 | (102) |
| **Hits** | .86 | (.07) | .85 | (.08) |
| **False Alarms** | .14 | (.05) | .14 | (.05) |
| **Sensitivity (*d’*)** | 2.22 | (.27) | 2.19 | (.39) |
| **Judgement Noise (SD)** | 4.16 | (1.76) | 4.61 | (1.87) |

1. ^1^ **Corresponding Author**: xavier.job@sorbonne-universite.fr [↑](#footnote-ref-1)
